# Resveratrol as a Natural Anti-Tumor Necrosis Factor-α Molecule: Implications to Dendritic Cells and Their Crosstalk with Mesenchymal Stromal Cells

**DOI:** 10.1371/journal.pone.0091406

**Published:** 2014-03-10

**Authors:** Andreia M. Silva, Marta I. Oliveira, Laura Sette, Catarina R. Almeida, Maria J. Oliveira, Mário A. Barbosa, Susana G. Santos

**Affiliations:** 1 INEB - Instituto de Engenharia Biomédica, Universidade do Porto, Porto, Portugal; 2 Faculdade de Engenharia, Universidade do Porto, Porto, Portugal; 3 Instituto de Ciências Biomédicas Abel Salazar, Universidade do Porto, Porto, Portugal; 4 Faculdade de Ciências, Universidade do Porto, Porto, Portugal; 5 Faculdade de Medicina, Universidade do Porto, Porto, Portugal; University of Bergen, Norway

## Abstract

Dendritic cells (DC) are promising targets for inducing tolerance in inflammatory conditions. Thus, this study aims to investigate the effects of the natural anti-inflammatory molecule resveratrol on human DC at phenotypic and functional levels, including their capacity to recruit mesenchymal stem/stromal cells (MSC). Primary human monocyte-derived DC and bone marrow MSC were used. DC immunophenotyping revealed that small doses of resveratrol (10 µM) reduce cell activation in response to tumor necrosis factor (TNF)-α, significantly decreasing surface expression of CD83 and CD86. Functionally, IL-12/IL-23 secretion induced by TNF-α was significantly reduced by resveratrol, while IL-10 levels increased. Resveratrol also inhibited T cell proliferation, in response to TNF-α-stimulated DC. The underlying mechanism was investigated by Western blot and imaging flow cytometry (ImageStream^X^), and likely involves impairment of nuclear translocation of the p65 NF-κB subunit. Importantly, results obtained demonstrate that DC are able to recruit MSC through extracellular matrix components, and that TNF-α impairs DC-mediated recruitment. Matrix metalloproteinases (MMP) produced by both cell populations were visualized by gelatin zymography. Finally, time-lapse microscopy analysis revealed a significant decrease on DC and MSC motility in co-cultures, indicating cell interaction, and TNF-α further decreased MSC motility, while resveratrol recovered it. Thus, the current study points out the potential of resveratrol as a natural anti-TNF-α drug, capable of modulating DC phenotype and function, as well as DC-mediated MSC recruitment.

## Introduction

Dendritic Cells (DC) are important in induction of adaptive immunity and promoting peripheral tolerance [1], [2]. Tumor Necrosis Factor **(**TNF)-α stimulates DC maturation, being used for induction of anti-tumor immunity [3], and promotes DC differentiation from monocytes [4]. However, TNF-α is also involved in chronic inflammatory processes, with several anti-TNF-α drugs being used as the most recent forms of treatment [5]. Moreover, differences in DC populations in rheumatoid arthritis and ankylosing spondylitis patients are reported [6], and the spondyloarthropathies rat model reveals DC with altered cytoskeletal dynamics, deficient motility and impaired DC–CD4 T cell immunological synapse formation [7], [8]. The main problems associated with anti-TNF-α treatments include long-term side effects, such as reduction of capacity to fight pathogens, no inflammation resolution and no reversal of existing joint damage. Also, relapse of inflammation when treatment is interrupted and high cost, constitute important drawbacks.

Treatments with the anti-inflammatory and anti-oxidant molecule resveratrol, a natural phytoalexin, have been shown to improve arthritic conditions in animal models [9], [10]. A recent study demonstrated that dietary supplementation of animal allergy models with resveratrol inhibits allergy development, possibly through DC involvement [11]. Resveratrol has been reported as interfering with TNF-α mRNA splicing in fish [12], and inhibiting TNF-α effects in some human cell lines [13], [14], [15]. A recent report indicates that human monocyte-derived DC treated with resveratrol gain tolerogenic properties, in response to LPS activation [16].

Thus, this work aimed at uncovering the potential of resveratrol to counteract effects of TNF-α on human DC, in terms of their phenotype and function, including their crosstalk with human mesenchymal stem/stromal cells (MSC). MSC are reported as immunomodulators, and can differentiate into bone and cartilage cells [17]. Also, MSC recruitment by macrophages and lymphocytes, such as Natural Killer (NK) cells [18] has been described.

Results reported here reveal that small doses of resveratrol are sufficient to inhibit phenotypic and functional TNF-α effects on DC. Significantly, our results also demonstrate DC-mediated MSC recruitment, and the negative influence of a pro-inflammatory environment on that process. These findings support resveratrol as a natural anti-TNF-α drug, and suggest new therapeutic opportunities for modulation of DC-mediated MSC recruitment, to improve current strategies of bone and cartilage regeneration.

## Materials and Methods

### Ethics Statement

All samples obtained and procedures performed were in agreement with the principles of the Declaration of Helsinki.

Monocytes were isolated from surplus buffy coats (BC) from healthy blood donors. These were kindly donated by Instituto Português do Sangue and Centro Hospitalar de São João (CHSJ), from Porto, Portugal, as part of an agreement with the Hematology service of the hospital. This is covered by the ethical approval of the service, under which blood donors give informed written consent for the byproducts of their blood collections to be used for research purposes. No information on age, sex or any identifying element was provided to the researchers, so all samples were analyzed anonymously.

MSC were obtained from discarded human bone marrow tissues from patients undergoing total hip arthroplasty (<50 years old, no known inflammatory diseases). Patients gave informed written consent for tissue use for research purposes and procedures were approved by the CHSJ Ethics Committee. All samples were analyzed anonymously.

### Primary DC Cultures

Monocytes were isolated from BC from healthy blood donors, as described [19]. Briefly, peripheral blood mononuclear cells (PBMC) were collected from centrifuged BC (20 min, 1200 g, room temperature (rt), without brake), and incubated with *RosetteSep* human monocyte enrichment kit (StemCell Technologies SARL, Grenoble, France), according to manufacturer’s instructions. The mixture was diluted 1∶1, with PBS supplemented with 2% FBS (heat inactivated, Lonza, Basel, Switzerland), layered over Histopaque-1077 (Sigma-Aldrich), and centrifuged as before. The enriched monocyte layer was collected and washed with PBS. Population purity was evaluated and over 70% of the cells were found to be CD14^+^ [19]. Cells were plated at 1×10^6^ cells/ml in complete culture media (RPMI-1640+Glutamax, with 10% heat inactivated FBS and 1% penicillin G-streptomycin (P/S, Invitrogen, Paisley, UK)), further supplemented with 50 ng/ml IL-4 and GM-CSF (Immunotools, Friesoythe, Germany), and maintained in a humidified incubator, at 37°C and with 5%CO_2_. Monocyte-derived DC were differentiated for 4–5 days, to obtain immature DC [20], [21], and treated with 10 µM resveratrol (Sigma), as indicated, for 4 h before stimulation with 100 ng/ml of TNF-α (Immunotools). Controls were left unstimulated or treated with either resveratrol or TNF-α alone. LPS (50 ng/ml) was used as positive maturation control [22], [23].

### Primary MSC Isolation and Culture

MSC were isolated by density gradient centrifugation, followed by selection of adherent cells, and characterized as described [18]. After Lymphoprep (Axis-Shield, Norway) density gradient centrifugation (1100 g, 30 min, rt, without break), the layer of mononucleated cells was collected, plated at approximately 180000 cells/cm^2^ in MSC culture media (DMEM low glucose and Glutamax, supplemented with 10% heat inactivated MSC-qualified FBS and 1% P/S). Cells were maintained in a humidified incubator, at 37°C with 5%CO_2_ and after 72 h non-adherent cells were removed and fresh media was added. At 80% confluence cells were detached with 0.05% trypsin/EDTA (Invitrogen) and expanded. MSC isolation was confirmed by flow cytometry (cells were CD105, CD73 and CD90 positive, while CD45, CD34, CD14, CD19 and HLA-DR negative), and capacity to differentiate into osteoblasts, chondroblasts or adipocytes [18] (not shown). Assays were performed at passages 5–9.

### Flow Cytometry

Flow cytometry was performed as previously described [19]. Briefly, cells were washed and labeled (45 min, 4°C in the dark) in staining buffer (PBS, 2%FBS, 0.01%Azide), with the following antibodies: anti-human CD1a (PE, APC), anti-human CD3 (FITC, PE), anti-human CD11c (FITC), anti-human CD86-FITC, anti-human HLA-DR-PE (Immunotools), anti-human CD83-FITC (AbDSerotec, Kidlington, UK). Isotype and fluorochrome-matched control antibodies were used to define background staining. For cell viability, Annexin V-FITC staining was performed for 15 min, and followed by PI (propidium iodide, BD Biosciences) staining, just prior to acquisition. For each sample 10000 cells, gated according to forward and side scatter parameters, were analyzed using a FACS Calibur flow cytometer (BD Biosciences) with Cell Quest software. Results were analyzed using FlowJo software (TreeStar, Inc.). Mean fluorescence intensity (MFI) was calculated subtracting the respective isotype controls.

### Cytokine Production

Supernatants of DC cultures stimulated as indicated were collected and centrifuged (14000 rpm, 4°C), before being assayed for IL-12/IL-23 (p40 subunit), or IL-10 levels, by *enzyme*-*linked* immunosorbent assay (ELISA, Legend Max Human ELISA kits, BioLegend, CA, USA) according to manufacturer’s protocol. Sample concentrations (pg/ml) were determined from mean absorbance values for each set of samples, compared to a standard calibration curve.

### Analysis of T cell Proliferation

Monocytes were plated at 0.3×10^6^ cells per well, allowed to differentiate into DC as above and then stimulated as indicated. Enriched lymphocyte populations were obtained from BC of different donors. Briefly, BC were centrifuged over Lymphoprep (800 g, 30 min without brake), the PBMC fraction was collected, cells were washed with PBS and platted for 2 h, allowing monocytes to adhere. The non-adherent lymphocyte fraction was collected and lymphocytes were labeled at 1×10^7^ cells/ml, with 1 µM CFSE (Invitrogen), in PBS (37°C, 15 min), followed by two washes (5 min, 2500 rpm) in PBS with 20% FBS. Cells were suspended in complete culture media and 0.9×10^6^ lymphocytes were added to DC (3∶1 ratio) [19], [24], [25], or cultured alone with DC differentiating cytokines (IL-4 and GM-CSF), TNF-α or resveratrol. Phytohemagglutinin (PHA, Sigma-Aldrich) stimulation was used as positive proliferation control. After 7 days cells were harvested, surface labeled for CD3, and analyzed by flow cytometry, as above. T cell division was determined by the extent of CFSE halving, on CD3^+^ cells.

### Analysis of Nuclear Factor (NF)-κB Translocation by Imaging Flow Cytometry

Differentiated DC were stimulated for the indicated times, before harvesting, washing with ice-cold PBS and fixing in 4% paraformaldehyde (PFA) (15 min on ice). NF-κB was detected by immunostaining, with a monoclonal antibody for the human p65 subunit (Rockland). Staining was performed in PBS with 0.1% TritonX-100 and 2%FBS, for 20 min, followed by incubation with anti-mouse FITC-labeled secondary antibodies (Immunotools), in PBS with 2%FBS. Cell nuclei were stained with 20 µM DRAQ5 (Biostatus). Single stainings and negative controls were used for fluorescence compensation. Cells were filtered and acquired using an ImageStream^X^ imaging flow cytometer with INSPIRE software, and equipped with an Extended Depth of Field filter (Amnis, EMD Millipore), at the Bioimaging Center for Biomaterials and Regenerative Therapies (b.IMAGE). Data was analyzed with IDEAS software (Amnis, EMD Millipore). For each sample 15000 cells were acquired, and more than 5000 single, in focus, double positive for NF-κB and DRAQ5 cells were analyzed for NF-κB cytoplasmic or nuclear localization. The “Nuclear translocation wizard”, which determines the similarity between NF-κB and nuclear stainings, was used to calculate the similarity coefficient for each sample. Nuclear translocation was considered for similarity coefficients >1.

### Western Blotting

DC were stimulated with TNF-α in presence or absence of resveratrol, or the inhibitor of IκBα phosphorylation (BAY 11-7082, Calbiochem), for the indicated times. Cells were then harvested and washed twice with cold PBS, before cell lysis in presence of protease and phosphatase inhibitors. Lysates were centrifuged (14000 rpm, 10 min, 4°C) and protein was quantified, using Bradford reagent (Bio-Rad). The same amount of protein per sample was resolved by SDS-PAGE in reducing conditions, and transferred to nitrocellulose membranes, which were blocked with 5% nonfat dry milk in PBS-T (PBS with 0.1% Tween-20). Membranes were then probed with one of the following primary antibodies: anti-p65 (as above); anti-phospho-NF-κB p65 (Ser536); anti-phospho-p38; anti-phospho-ERK1/2; anti-phospho-JNK (all from Cell Signaling, UK); or anti-α-tubulin (Sigma-Aldrich). Appropriate secondary antibodies conjugated to streptavidin-HRP were used for signal detection, upon membrane incubation with chemiluminescent substrate (ECL, GE Healthcare) and exposure to X-ray films.

### MSC Recruitment Assays

Differentiated DC were harvested and 4×10^5^ cells were plated in MSC serum-free culture media. Cells were stimulated as indicated for 24 h prior to recruitment assays, performed as described [18]. Briefly, transwell inserts with 8 µm pore size membranes, coated with Matrigel (BD Biosciences) were placed into wells, creating a top compartment, where 4×10^4^ MSC (1MSC:10DC) were seeded in MSC serum-free media. Negative controls had no DC in bottom compartments. After 24 h the assay was stopped, culture media were collected for matrix metalloproteinases (MMP) analysis and insert membranes were washed with PBS, fixed with 4%PFA, and washed again. Non-invading cells were removed by scrubbing the membranes inner side with a cotton swab. Membranes were then removed, mounted on a microscope slide, using vectashield with DAPI (4′,6-diamidino-2-phenylindole, Vector Laboratories) and visualized through a Zeiss Axiovert200 fluorescence microscope (Carl Zeiss, Germany). Total number of recruited MSC was counted, as the number of nuclei that completely passed through filter pores.

### Gelatin Zymography

Culture media from recruitment experiments was collected from top and bottom compartments, separately. Samples were centrifuged (1200 rpm, 10 min, 4°C), and proteins in supernatants were precipitated with acetone (1∶6, vol:vol) at −20°C, before centrifuging (14000 rpm, 5 min, 4°C) and discarding supernatants. Acetone remnants were evaporated at rt, protein pellets dissolved in 30 µl ultrapure water under agitation and protein content was quantified using *Dc* Protein Assay (Bio-Rad). Samples were prepared by mixing 2.25 µg of protein per sample with loading buffer (SDS 0.1%, 0.04% sucrose in Tris buffer 0.25 M, pH 6.8) and resolved in zymography gels: stacking gels-5% polyacrylamide (Bio-Rad); resolving gels-10% polyacrylamide containing 0.1% gelatin (Sigma-Aldrich), both with 0,1% SDS. Gels were incubated twice in 2% TritonX-100 (Sigma-Aldrich) for 15 min, rinsed with water and incubated in MMP substrate buffer (CaCl_2_ 10 mM in Tris buffer 50 mM, pH7.5) for 16–18 h (37°C, under agitation). After, gels were rinsed with water, incubated with Coomassie Brilliant Blue R-250 solution (Sigma) in 50% methanol and 10% acetic acid (Merck, Algés, Portugal) and washed with water until clear proteolytic activity bands were seen against the Coomassie-stained, gelatin blue background.

### Time-lapse Video-microscopy

Co-cultures were prepared by plating 5×10^3^ MSC per well on a 24-well plate (in MSC complete culture media), 24 h before adding DC at 1MSC:10DC ratio, in fresh MSC culture media. MSC or DC single cultures were also prepared. The plate was then imaged using a microscope with incubation system (Zeiss Axiovert200), at 37°C/5%CO_2_. Cell positions were defined and phase contrast photographs recorded every 5 min, for 12 h. Videos were analyzed with MTrackJ (ImageJ) for single cell tracking.

### Statistical Analysis

Prism5 software, vs5.0a was used. Gaussian distribution was tested with D’Agostino&Pearson test, and samples were found to be non-parametric. Wilcoxon or Mann-Whitney tests were used to compare two samples, whereas Friedman or Kruskal-Wallis followed by Dunns multiple comparison test were chosen for multiple comparisons. A value of p<0.05 was considered statistically significant.

## Results

### A Small Dose of Resveratrol is Sufficient for Reducing DC Activation

**Figure 1:**
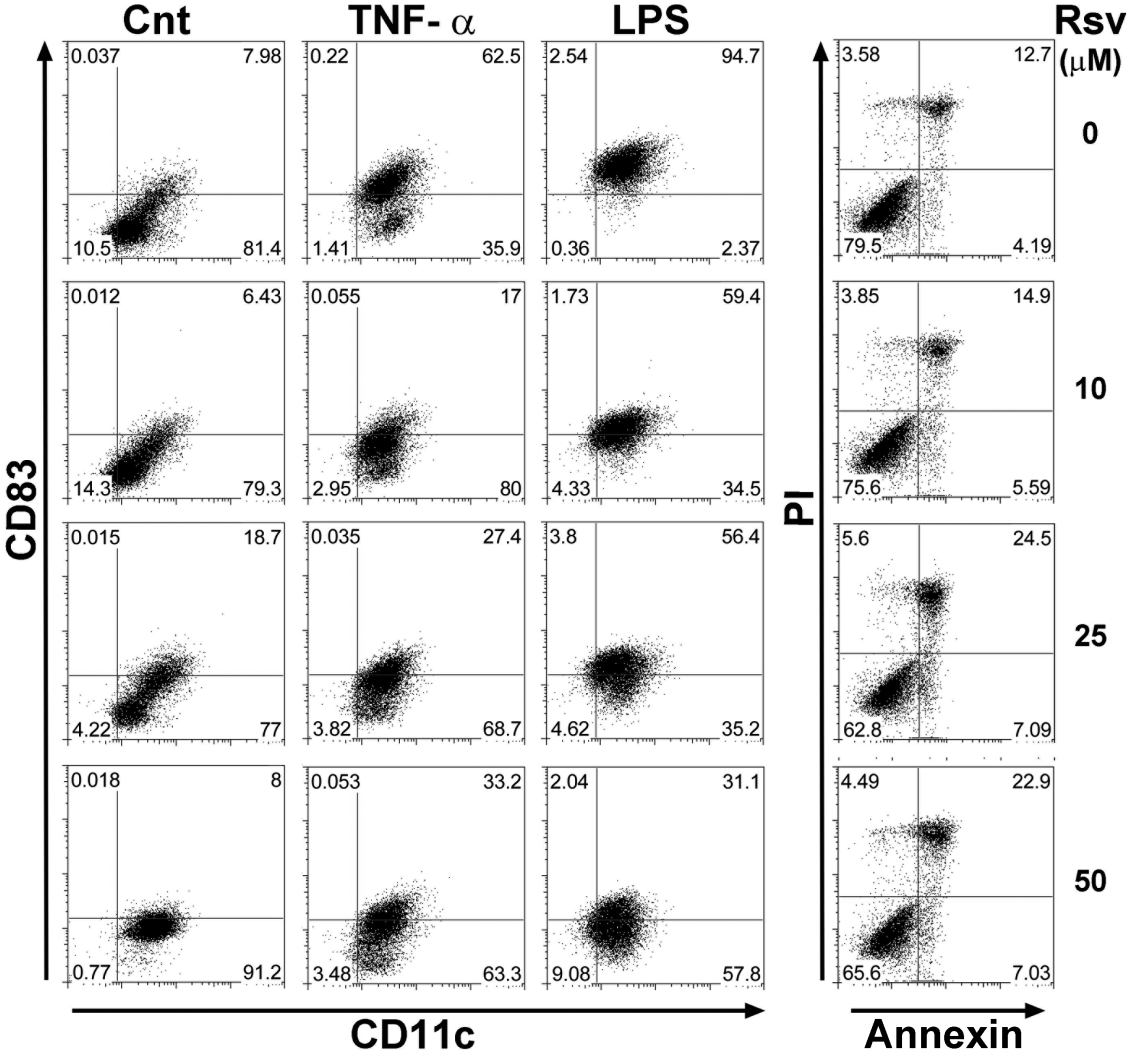
Resveratrol inhibits DC stimulation without significant cell death. Differentiated DC were treated with 10, 25 or 50 µM resveratrol prior to stimulation with TNF-α or LPS. Cells were surface stained for lineage marker CD11c and activation marker CD83 (left panel), or AnnexinV and PI (right panel). Samples were analyzed by flow cytometry. Values on each quadrant represent percentage of cells in that quadrant (Cnt: control, rsv: resveratrol). Data is representative of three experiments.

In order to investigate the potential influence of resveratrol treatment under pro-inflammatory conditions, we started by establishing the working resveratrol concentration. Immature DC were stimulated with TNF-α or LPS, in presence or absence of resveratrol. Results illustrated in Figure 1 show that cells were positive for DC lineage marker CD11c, and up-regulated the cell surface activation marker CD83, upon TNF-α stimulation, albeit to lower levels than after LPS stimulation. All concentrations of resveratrol tested were able to reduce DC activation, under TNF-α or LPS stimulation. Interestingly, a concentration of just 10 µM of resveratrol was able to considerably reduce CD83 cell surface levels, particularly under TNF-α stimulation (Figure 1). Toxicity of resveratrol was evaluated, by measuring cell death and apoptosis, with Annexin V and PI staining. Results obtained show an increase in apoptosis as resveratrol concentration increases, but 10 µM did not induce apoptosis above control levels (Figure 1). Therefore, 10 µM was the concentration selected for subsequent experiments.

### Resveratrol Inhibits DC Maturation Phenotype and Modulates Cytokine Secretion in Simulated Pro-inflammatory Conditions

**Figure 2:**
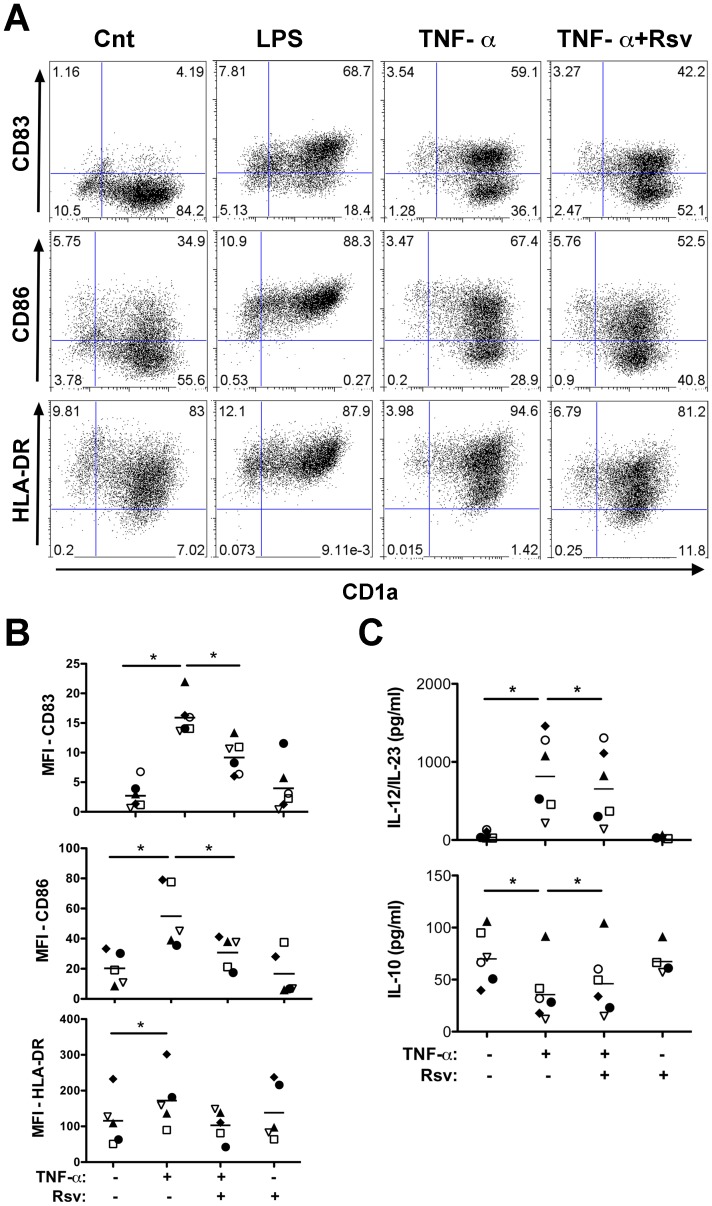
Resveratrol inhibits TNF-α-stimulation on DC. Differentiated DC were stimulated as indicated, with LPS or TNF-α, in presence or absence of resveratrol for 72 h, before surface staining for CD1a and: CD83 (top row), or CD86 (middle row) or HLA-DR (bottom row). Samples were analyzed by flow cytometry. **A.** Plots of a representative phenotypic profile, with values in each quadrant representing percentage of cells in that quadrant. **B.** Graphs represent isotype control-subtracted mean fluorescent intensity (MFI) of CD83 (top graph), CD86 (middle graph) or HLA-DR (bottom graph), over 6 independent experiments. **C.** Cell culture supernatants were collected and analyzed for the indicated cytokines by ELISA. Each donor is represented as a different symbol and the mean value, over 6 independent experiments, is represented as a dash. Statistical significance was calculated using the Wilcoxon signed rank test (*p<0.05).

To further understand the influence of resveratrol on DC response to TNF-α, cell phenotype was investigated. Results obtained demonstrate that DC do not loose the expression of the lineage marker CD1a, but specifically decrease cell surface expression of CD83, CD86 and HLA-DR, induced by exposure to TNF-α (Figure 2A). Quantification of results across different donors, shown in Figure 2B, evidenced a significant increase for CD83, CD86 and HLA-DR expression on CD1a positive cells, in response to TNF-α. Moreover, a significant MFI decrease for CD83 and CD86 was found in presence of resveratrol, indicating inhibition of DC activation. Down-modulation of HLA-DR was somewhat more modest, but followed the same trend.

DC activation leads to cytokine production, which is dependent on the activation stimulus, and conditions T cell activation and polarization. Thus, we analyzed DC production of the common subunit of two very important pro-inflammatory members of the IL-12 family of cytokines, IL-12 and IL-23, and also the anti-inflammatory cytokine IL-10, under TNF-α stimulation, alone or in presence of resveratrol. Results illustrated in Figure 2C show that TNF-α stimulation leads to significant increase of IL-12/IL-23, and decrease of IL-10 levels. Importantly, the presence of resveratrol altered DC cytokine production, significantly decreasing IL-12/IL-23 and increasing IL-10 secretion, relative to TNF-α stimulation alone. Thus, results presented this far show important modulation of DC maturation phenotype and cytokine secretion by resveratrol.

### Resveratrol Inhibits DC-induced T Cell Proliferation

**Figure 3:**
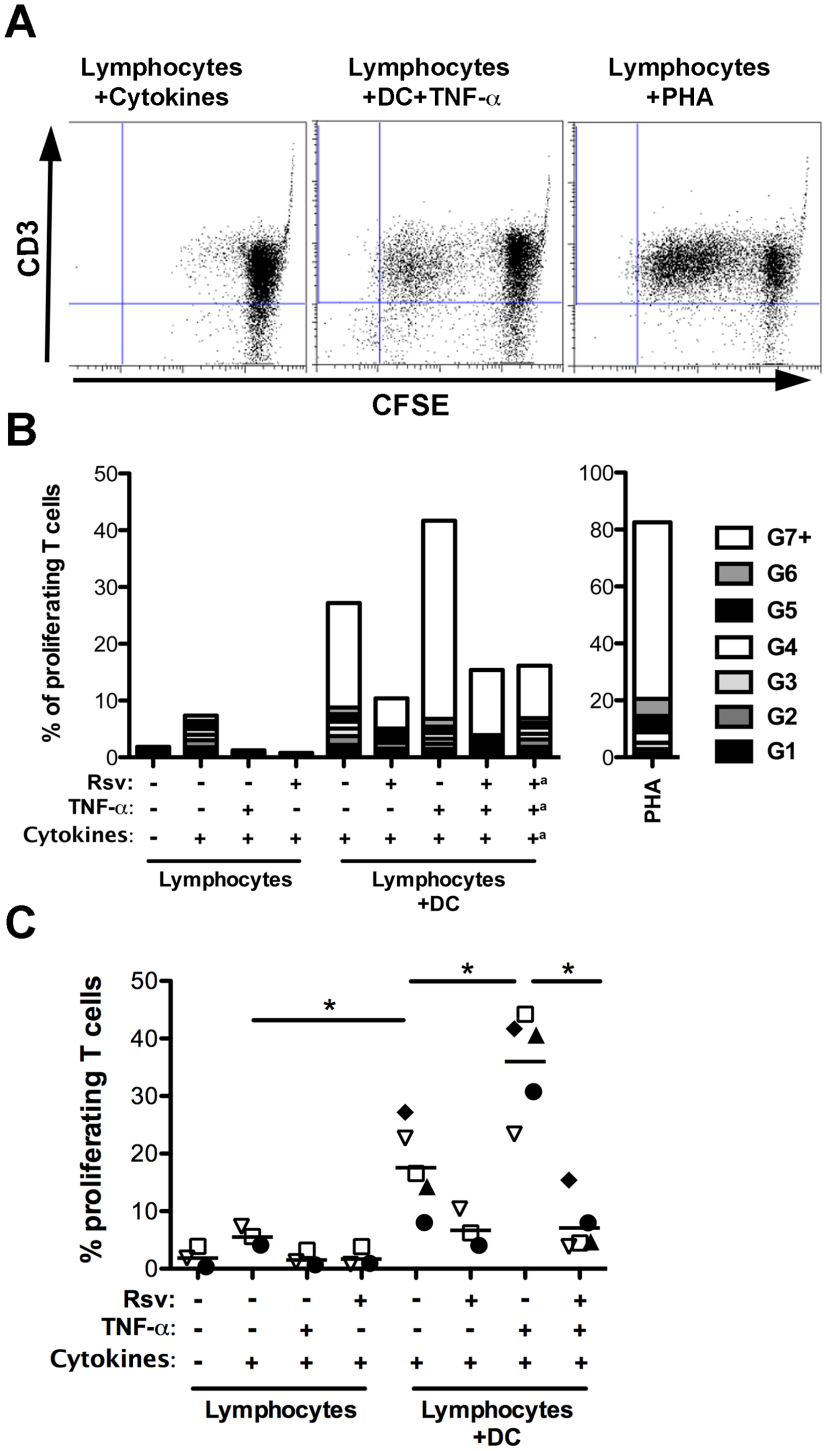
Resveratrol inhibits T cell proliferation in response to TNF-α stimulated DC. Differentiated DC were stimulated with TNF-α, in presence or absence of resveratrol as indicated, for 24 h, before adding CFSE-labeled lymphocytes from a different individual. Lymphocytes cultured with DC differentiating cytokines (GM-CSF and IL-4), PHA, TNF-α alone or in presence of resveratrol were used as controls. After 7 days cells were stained for CD3 and analyzed by flow cytometry. **A.** Representative plots of lymphocytes cultured: alone with DC differentiating cytokines (left), with TNF-α-stimulated DC (middle), and alone stimulated with PHA (right). The CD3 positive population was gated for further analysis. **B.** Representative plot of the percentage of T lymphocytes with different numbers of cell divisions (generations - G), with ^a^ representing samples where TNF-α and resveratrol were removed before addition of lymphocytes. **C.** Quantification of the percentage of proliferating T cells for 5 independent experiments. Each experiment is represented as a different symbol and a dash represents the mean. Comparisons were performed using the Kruskal-Wallis followed by Dunns multiple comparisons test (* p<0.05).

In order to investigate the influence of resveratrol on DC function, DC-induced T cell proliferation was evaluated. Differentiated DC were stimulated with TNF-α in presence or absence of resveratrol and then cultured with CFSE-labeled lymphocytes from a different donor, setting up a mixed lymphocyte reaction. In agreement with previous reports [16], [19], the results obtained show that monocyte-derived DC had the capacity of inducing proliferation of T cells (Figure 3A and B). As expected, the percentage of T cells that responded to DC increased when DC were stimulated with TNF-α, when compared to unstimulated DC (Figure 3B). Interestingly, resveratrol reduced the percentage of T cells that divided in response to DC (Figure 3B). To exclude the possibility that resveratrol presence in cultures could preclude T cell proliferation independently of DC, samples were obtained where culture media was changed, removing resveratrol, before adding the lymphocytes. Results were found to be similar to those without media change (Figure 3B), indicating an effect of resveratrol on DC-induced T cell proliferation. Lymphocytes cultured with resveratrol, TNF-α or GM-CSF and IL-4 were used as negative controls, whereas PHA stimulation was the positive control.

When T cell proliferation was quantified over several independent experiments (Figure 3C), significant increases were observed in presence of DC versus differentiating cytokines alone, and further in presence of DC activated with TNF-α, as expected. Notably, a significant reduction of proliferation was found in response to DC that had been stimulated with TNF-α in presence of resveratrol, when compared to proliferation induced by TNF-α stimulated DC (Figure 3C).

### NF-κB Nuclear Translocation in Response to TNF-α is Impaired in Presence of Resveratrol

**Figure 4:**
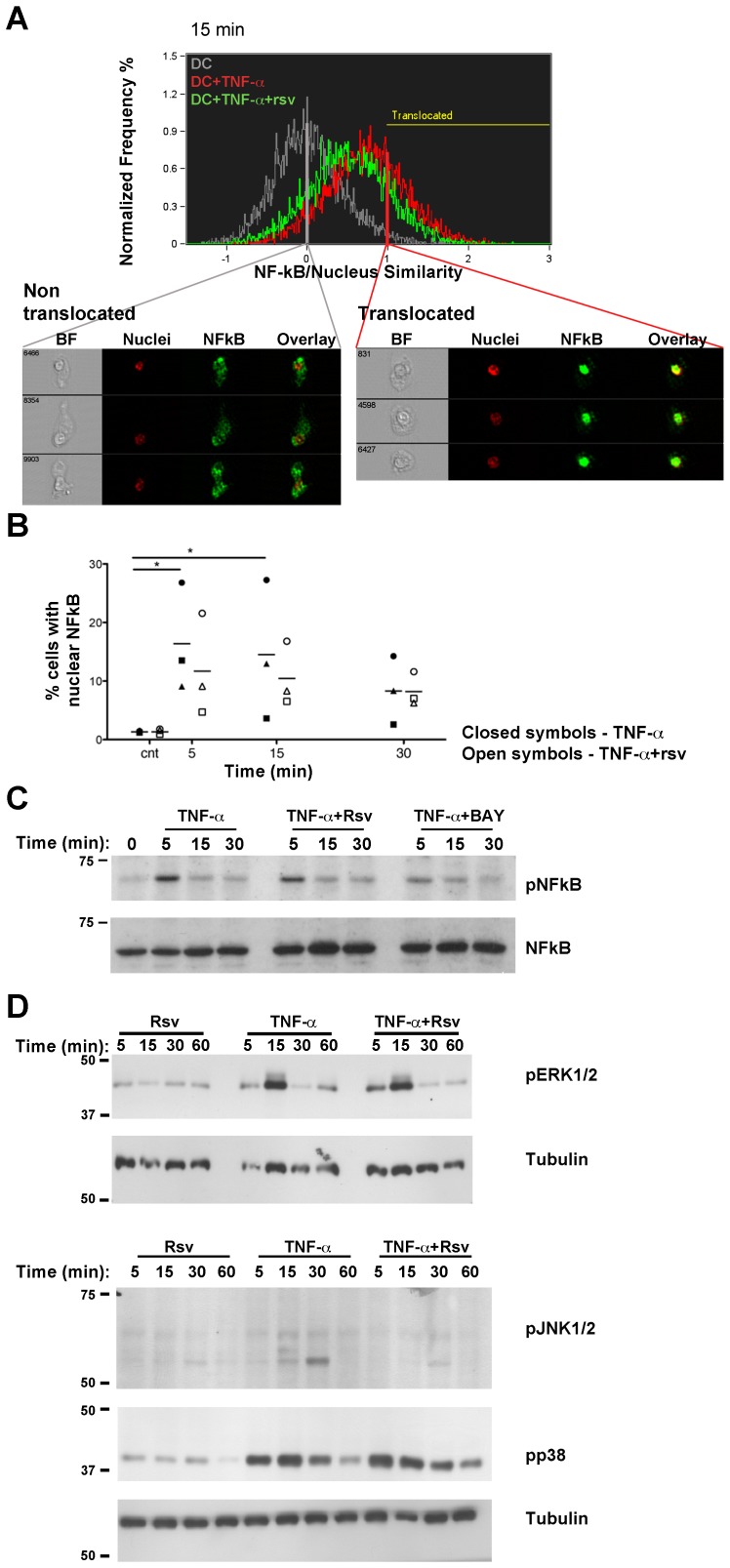
Resveratrol inhibits NF-κB signaling in DC exposed to TNF-α. DC were stimulated with TNF-α, in presence or absence of resveratrol, for the indicated times. **A.** Cells were stained for NF-κB (p65 subunit, green staining) and nuclei were counterstained with DRAQ5 (red staining), before acquisition by ImageStream^X^. Representative plot of the similarity coefficient between nucleus and NF-κB stainings of unstimulated DC (grey), DC stimulated with TNF-α alone (red), or in presence of resveratrol (Rsv, green). The yellow line corresponds to the gate for which nuclear translocation of NF-κB was considered. Three representative images of cells found in the indicated areas of the graph are shown. For the control condition, cells with a 0 similarity coefficient (no p65 translocation, grey line), and for the TNF-α -stimulated condition, cells with a similarity coefficient of 1 (NF-κB nuclear translocation, red line) are shown. BF: brightfield. **B.** Quantification of the percentage of cells with nuclear NF-κB along time (within the translocated gate) for different donors. Each symbol represents a different individual (the mean value is represented as a dash), and TNF-α stimulation in absence or presence of resveratrol is represented by closed or open symbols, respectively. Comparisons were performed using Friedman test, followed by Dunns multiple comparisons test (* p<0.05). **C.** Phosphorylation of p65 in response to TNF-α, in presence or absence of resveratrol or BAY 11-7082 (BAY) and **D.** MAPK signaling in response to TNF-α and/or resveratrol was evaluated by Western blot. DC were stimulated with TNF-α, in presence or absence of resveratrol, for indicated times. Cells were lysed and samples resolved in reducing gels, which were blotted onto nitrocellulose membranes, and probed with the indicated antibodies. The images are representative of three independent experiments. p: phosphorylated.

Aiming at elucidating the signaling mechanisms underlying the changes in DC phenotype and function observed above, we next investigated the involvement of the NF-κB signaling mediator, previously described as a target of resveratrol [16], [26], [27], as well as mitogen activated protein kinases (MAPK) signaling pathways. Taking advantage of the potential of imaging flow cytometry, NF-κB subunit p65 (RelA) nuclear translocation was analyzed using ImageStream^X^. By combining the flow cytometry advantage of analyzing a high number of cells, with the spatial resolution of microscopy imaging, this technique allows the visualization of intracellular localization of stained molecules, for each acquired cell. Figure 4A shows a representative merged histogram of the NF-κB/nucleus similarity coefficient, for DC that were left untreated (grey line), or stimulated with TNF-α, in absence (red line) or presence (green line) of resveratrol. Also, to illustrate how the translocation gate was designated, representative cells with a similarity coefficient of 0, where NF-κB did not translocate to the cell nucleus (non-translocated) are shown for the control condition (grey line), and representative cells with a similarity coefficient of 1, where NF-κB was translocated to the cell nucleus, are shown for the TNF-α stimulated sample (red line). For each sample, the extent of the histogram curve that is beyond the similarity coefficient of 1 determined the percentage of cells that show NF-κB nuclear translocation. Results for three independent experiments show significant NF-κB nuclear translocation at 5 and 15 min upon TNF-α stimulation (Figure 4B, closed symbols) and a decrease after 30 min. Different individuals showed different kinetics for NF-κB translocation. Nonetheless, presence of resveratrol (open symbols) partially inhibited NF-κB translocation for every donor, particularly at the time-point(s) when it was maximum. To further confirm these results the phosphorylation of the p65 NF-κB subunit was also evaluated (Figure 4C). The results show maximal p65 phosphorylation at 5 min and partial inhibition by the presence of resveratrol. The specific inhibitor of TNF-α-inducible phosphorylation of IκBα, BAY 11-7082 (BAY), was also used as a positive control. As expected, BAY led to increased inhibition of p65 phosphorylation in response to TNF-α. Finally, the three MAPK pathways were also probed, by Western blot analysis of the phosphorylated forms of ERK1/2, p38 and JNK. Although phosphorylation of all pathways occurred in response to TNF-α, the presence of resveratrol did not consistently inhibit it (Figure 4D).

### TNF-α Impairs DC Capacity to Recruit MSC, While Resveratrol Rescues MSC Motility

**Figure 5:**
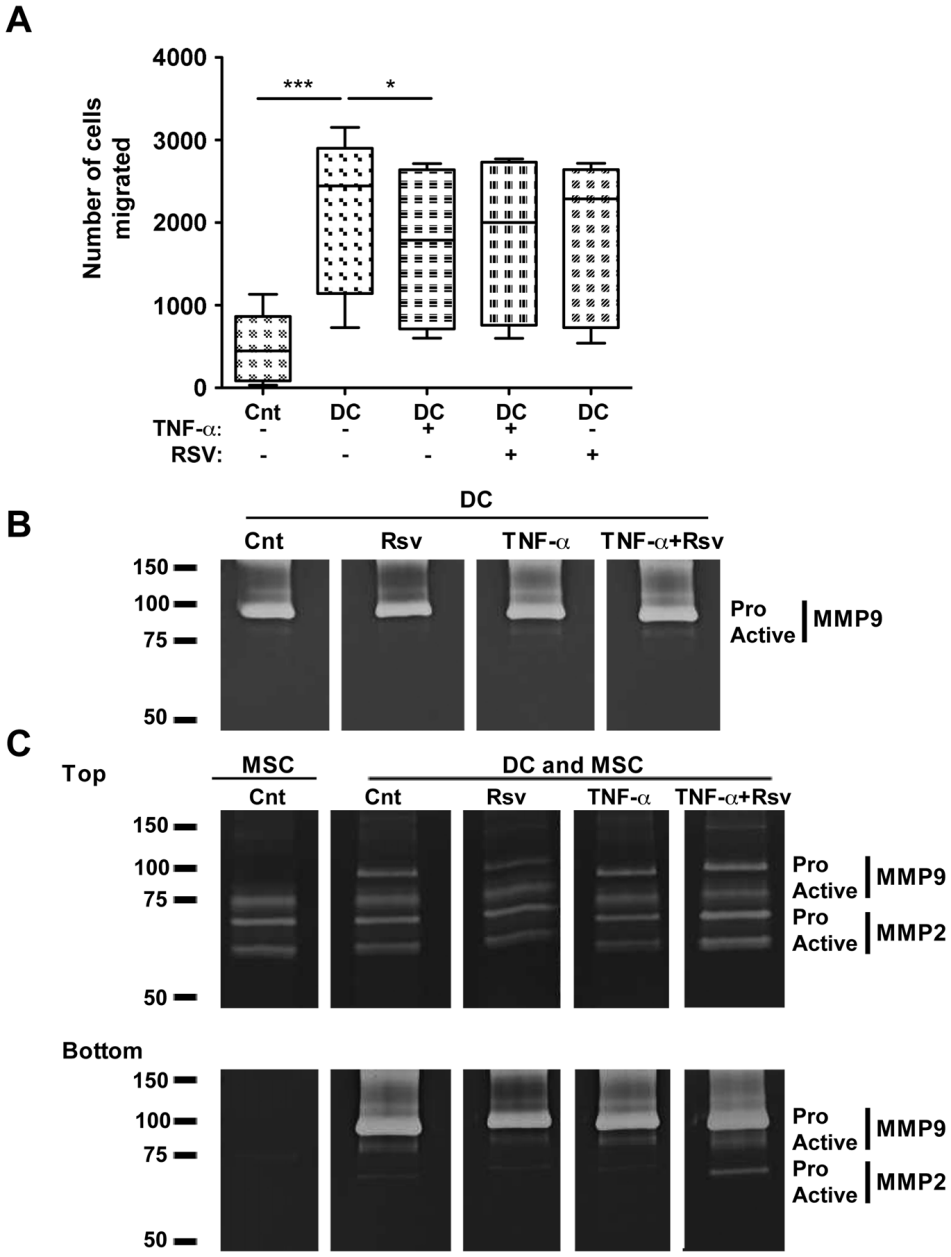
TNF-α impairs DC-mediated MSC recruitment. Differentiated DC were stimulated with TNF-α in presence or absence of resveratrol, as indicated. **A.** Matrigel-coated transwell inserts were introduced on top of these wells and MSC were seeded in the upper compartment, at a ratio of 1MSC:10DC. MSC recruitment was allowed to take place for 24 h. MSC on the bottom of the filter were stained with DAPI and all migrated cells counted under a fluorescence microscope. The Box&Whiskers plot represents all values (min-to-max), and the median (dash) for each condition, across 6 different DC donors. Cnt – control conditions without DC in the bottom compartment. Comparisons were performed using Friedman test, followed by Dunns multiple comparisons test (* p<0.05, *** p<0.001). **B.** Culture supernatants from DC alone or from **C**. Top and Bottom compartments of transwell experiments described above, were collected, their protein content quantified and then resolved by gelatin zymography. Proteolytic white bands were revealed on a Coomassie Blue-stained background.

Since bone regeneration is impaired in several inflammatory conditions, we next assessed DC capacity to recruit MSC, and how that could be influenced by pro- *versus* anti-inflammatory DC stimulation. Human MSC used in this study were isolated from bone marrow and characterized to follow the criteria of the international society for cellular therapy [18], [28] (not shown). Results show that DC have a significant ability to recruit MSC (Figure 5A). Initially, three different DC:MSC ratios were tested, and an increase in MSC recruitment was observed from 5∶1 to 10∶1 ratios, but no further increase at 20∶1 ratio (not shown), so 10∶1 ratio was selected for this study. Stimulation of DC with TNF-α significantly impaired their ability to recruit MSC, while presence of resveratrol led to a tendency for the recovery of MSC recruitment, albeit not statistically significant (Figure 5A).

MMP are reported to have a role in extracellular matrix remodeling, promoting cell migration. Thus, supernatants from recruitment assays were used to analyze MMP activity. Results illustrated in Figure 5B show DC as producers of pro-MMP9, but when cultured alone the active form of the enzyme could hardly be detected (Figure 5B). When the two cell populations were cultured separated by transwell inserts, the top and bottom compartments were analyzed separately (Figure 5C). Although during the assay media will likely be exchanged between the compartments, they were analyzed separately to get an indication of which cell population could be the main producer of each MMP. Results illustrated in Figure 5C indicate that MSC alone did not produce detectable amounts of pro-MMP9, but when DC were present, the active form of MMP9 could be observed in top and bottom compartments. No consistent differences were found for pro or active forms of MMP9 or MMP2 under the influence of TNF-α, alone or in presence of resveratrol.

**Figure 6:**
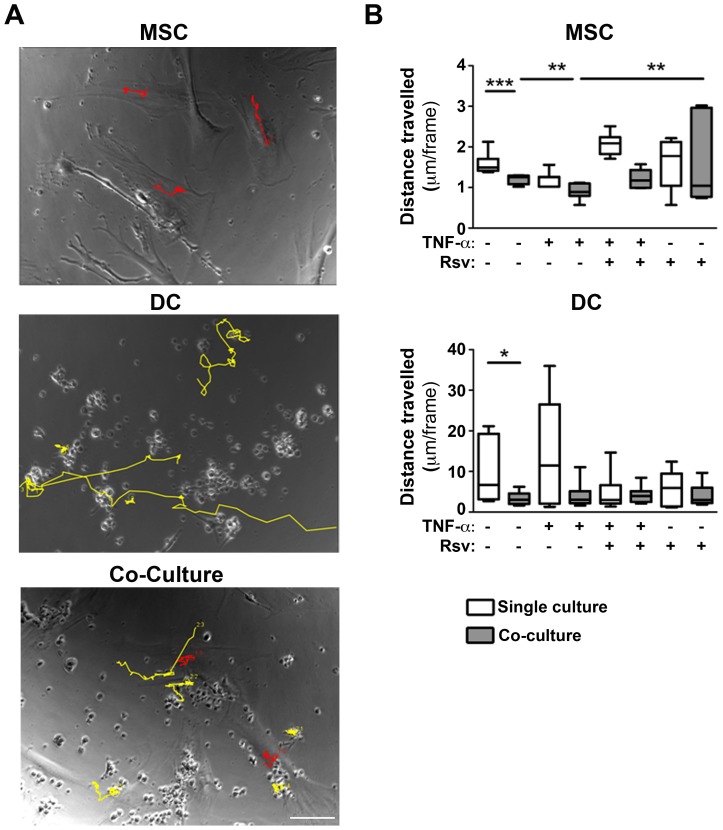
Presence of resveratrol rescues MSC motility in co-cultures with DC. DC were differentiated and stimulated as indicated, before being added to MSC cultures (1MSC:10DC). The cultures were followed by time-lapse video-microscopy for 12 h. Image analysis was performed using ImageJ software. **A**. Images with representative pathways from cell tracking using ImageJ. Examples of MSC (red) and DC (yellow) tracks are highlighted. Scale bar: 100 µm. **B**. Quantification of MSC and DC travelling distance when cultured together, *versus* separately. Box&Whiskers plots represent median (dash) and range of values. Statistical significance was calculated using Mann-Whitney test (*p<0.05, **p<0.01, ***p<0.001).

Finally, in order to further investigate the recruitment impairment observed in the presence of TNF-α, we investigated DC and MSC motility. Figure 6A illustrates an example of the tracking performed for MSC (red) and DC (yellow). A significant decrease in MSC and DC motility in co-culture, when compared to monoculture, was observed (Figure 6B). Interestingly, a further decrease in MSC motility was found when they were co-cultured with TNF-α stimulated DC, and a significant recovery of motility was observed in presence of resveratrol (Figure 6B), which supports the results obtained for MSC recruitment.

## Discussion

Resveratrol has been reported as an anti-inflammatory molecule, able to inhibit TNF-α effects in different cell populations [15], [27], [29], [30], [31], as well as LPS-induced DC activation [16], [32], and to ameliorate inflammatory arthritic conditions in animal models [9], [10]. In the current study, we investigated phenotypic and functional responses of primary human DC exposed to TNF-α alone, or in presence of resveratrol, and analyzed for the first time its consequences for DC-mediated MSC recruitment.

Envisaging a future clinical application we sought to define the lowest resveratrol concentration capable of having an effect on DC. Dietary resveratrol is obtained mainly from grapes, wine, peanuts and their derivatives. Its concentration in different wines has been reported to range from 0.99****µmol/L to 25.49 µmol/L [33]. Nevertheless, as its bioavailability is quite low, it is not likely that resveratrol normally ingested in diet would be available for cells in the concentrations previously shown to have biological activity in *in vitro* assays (5–100 µmol/L) [34]. So, high doses of resveratrol would have to be obtained as a dietary supplement, and ideally encapsulated in an adequate delivery system that could ensure good pharmacokinetics and bioavailability at cellular level on target sites [35], [36]. Conversely, a concentration of 50 µM of resveratrol was recently reported as non-toxic for DC [16]. The results herein presented indicate a small increase of apoptotic cells, when increasing concentrations of resveratrol were used. Beyond previous reports on the inhibition of DC maturation in response to LPS [16], [32], we found that resveratrol, even in small concentrations, was able to inhibit the effects of DC exposure to TNF-α. Also, adding resveratrol just hours prior to TNF-α stimulation was enough to observe significant inhibition of CD83 and CD86 up-regulation, which did not recover, even at 72 h post-stimulation. This is not in full agreement with a previous report indicating the need to add resveratrol during DC differentiation to observe a lasting inhibition of CD83 up-regulation [16]. The authors also reported a reduction of CD1a expression [16], indicating reduced differentiation or de-differentiation of DC. Here, two different lineage markers, (CD11c and CD1a) confirmed no DC de-differentiation upon incubation with resveratrol for up to 72 h. Importantly, differences observed on cell surface expression of phenotypic markers were further reflected in the change of cytokine profile, demonstrating that TNF-α was inducing DC activation and that resveratrol can inhibit maturation under inflammatory conditions.

The mechanism of action for resveratrol is a topic of intense debate and many pathways were previously reported as targets [13], [16], [26], [27], [37], [38], [39]. The pathway targeted by resveratrol will likely depend on the cell population and stimuli being studied. Thus, for inflammatory diseases where no bacterial culprit has been identified it is important to study the effect of pro-inflammatory mediators involved, such as TNF-α, instead of the commonly used LPS stimulation. In order to investigate the molecular mechanism of resveratrol action on human primary DC in simulated pro-inflammatory conditions we probed different signaling pathways. NF-κB is a reported resveratrol target in different cell lines [26] and in DC [16]. NF-κB p65 phosphorylation, followed by translocation to the cell nucleus reflects activation of this pathway, which will lead to the activation of transcription of target genes. In the work here presented NF-κB pathway was activated in response to TNF-α, and notably, the presence of resveratrol led to a decrease in p65 phosphorylation and translocation. The results obtained by imaging flow cytometry show a common tendency amongst all donors tested, but are not statistically significantly different. This is likely related to the fact that NF-κB signaling is maximum at different time-points (5 or 15 min) and different levels, depending on the DC donor, somewhat impairing the statistical comparison. Nonetheless, the results presented for translocation and phosphorylation of p65 together show an impairment of TNF-α-induced NF-κB signaling in presence of resveratrol. Thus, our results suggest that the NF-κB pathway reported to be involved for LPS stimulation of primary human DC [16], may also be important in the case of TNF-α stimulation, indicating that resveratrol acts on DC primarily by inhibiting NF-κB activation.

Our main interest was in investigating functional consequences of resveratrol, which may have implications for inflammatory diseases. As T cell activation plays important roles in disease, capacity of DC to stimulate allogeneic T lymphocytes was evaluated. In agreement with a previous report on LPS-stimulated DC [16] resveratrol was also able to reduce the percentage of T lymphocytes that responded to DC, both immature and TNF-α-stimulated. Resveratrol is reported to inhibit cancer cell proliferation [40] and in our assay the presence of resveratrol, in the absence of TNF-α, led to a reduction of DC-induced T cell proliferation. So, we investigated if resveratrol presence, independently of TNF-α stimulation, would inhibit T cell proliferation, which was not the case. This indicates that the action of resveratrol will likely be through its effect on DC.

Having characterized DC response to TNF-α in presence or absence of resveratrol, both phenotypically and functionally, we were interested in its consequences for MSC recruitment. MSC are reported to have immunosuppressive properties [41], [42], [43], that may aid in controlling chronic inflammation and also are able to differentiate into bone and cartilage cells (osteoblasts and chondrocytes) [17], which could contribute to regeneration of bone and cartilage. MSC recruitment by immune cells has been reported, with macrophages being more potent recruiters than NK or T lymphocytes [18]. In this study we demonstrate for the first time that DC can recruit MSC, and a chemotatic index (relation between recruitment of MSC in the presence of DC *versus* negative control conditions) around 10 indicates that DC recruit MSC at higher levels than lymphocytes, close to what was observed for macrophages [18]. Immunosuppressive features of MSC have been extensively reported [17], including their capacity to modulate DC differentiation and maturation [44]. On the other hand, the influence of immune cells on MSC recruitment and differentiation has only recently started to be uncovered. These effects are important for bone regeneration in particular, but also for immunosuppression, as it is known that MSC are not immunosuppressive *per se* and instead exposure to pro-inflammatory cytokines is necessary to promote, or “license” MSC immunosuppressive activity [44].

DC-MSC interaction in co-cultures, observable in time-lapse data, is illustrated by a significant reduction of DC and MSC motility, when compared to single cultures. Interestingly, TNF-α impaired DC-mediated MSC recruitment was supported by a significant reduction of MSC motility in co-cultures, which was recovered in presence of resveratrol.

Our results are not in agreement with previous studies indicating that TNF-α promotes MSC migration [45], [46]. However, important differences may explain the diverging results, as both studies report on TNF-α as the sole factor of recruitment, while in the present work DC-MSC cell interaction in presence of TNF-α, alone or together with resveratrol, was considered. Also, Fu and colleagues used transwell inserts with polycarbonate membranes without any coating, and thus the assay simulates only migration [45], while Bocker and co-workers used extracellular matrix coated inserts [46], which is more comparable to this study. MSC migration through the basement membrane has been correlated with production of MMP, with MSC particularly producing MMP2 [47]. In our assays DC produce high amounts of pro-MMP9, which is virtually absent from MSC cultures and from top compartments of transwell recruitment assay media, but may aid MSC migration through Matrigel-coated inserts, in response to DC. A previous report indicated that resveratrol presence inhibits pro-MMP9 expression [48]. Nonetheless, gelatin zymography data did not reveal consistent differences in MMP9 production between DC unstimulated or stimulated with TNF-α, in presence or absence of resveratrol.

In conclusion, the results presented here indicate that resveratrol is able to inhibit the effects of TNF-α exposure on DC, in terms of cell phenotype, cytokine secretion, and their ability to stimulate T cells and to recruit MSC through extracellular matrix components. Moreover, the mechanism of resveratrol inhibition is likely mediated by NF-κB signaling. Finally, using resveratrol for targeting DC-MSC interface could present further benefits to bone and cartilage regeneration, as resveratrol has been previously reported to enhance proliferation and osteoblastic differentiation of MSC [37]. Taken together, our data suggests that resveratrol is a promising natural antagonist of TNF-α effects on DC, modulating DC-mediated MSC recruitment, thus enlarging its potential therapeutic applications.
